# Function-preserving radical surgery for early-stage non-small cell lung cancer: A review of limited resection approaches

**DOI:** 10.1093/jjco/hyag013

**Published:** 2026-02-05

**Authors:** Yoichi Ohtaki, Keiju Aokage, Tomohiro Miyoshi, Kenta Tane, Yuki Matsumura, Masahiro Tsuboi

**Affiliations:** Division of Thoracic Surgery, National Cancer Center Hospital East, 6-5-1 Kashiwanoha, Kashiwa, Chiba 277-8577, Japan; Division of Thoracic Surgery, National Cancer Center Hospital East, 6-5-1 Kashiwanoha, Kashiwa, Chiba 277-8577, Japan; Division of Thoracic Surgery, National Cancer Center Hospital East, 6-5-1 Kashiwanoha, Kashiwa, Chiba 277-8577, Japan; Division of Thoracic Surgery, National Cancer Center Hospital East, 6-5-1 Kashiwanoha, Kashiwa, Chiba 277-8577, Japan; Division of Thoracic Surgery, National Cancer Center Hospital East, 6-5-1 Kashiwanoha, Kashiwa, Chiba 277-8577, Japan; Division of Thoracic Surgery, National Cancer Center Hospital East, 6-5-1 Kashiwanoha, Kashiwa, Chiba 277-8577, Japan

**Keywords:** Lung cancer, segmentectomy, sublobar resection, ground glass opacity (GGO), consolidation tumor ratio (CTR)

## Abstract

Radical lobectomy, proposed as a curative treatment for lung cancer in 1960, has long been regarded as the standard surgical approach. The findings of two phase III randomized controlled trials comparing limited resection versus lobectomy for non-small cell lung cancer (NSCLC) ≤2 cm have challenged the long-standing evidence supporting lobectomy as the universal surgical option for all patients with lung cancer. The Japanese clinical oncology group (JCOG) and West Japan Oncology Group (WJOG) (JCOG0802/WJOG4607L) demonstrated both the non-inferiority and superiority of segmentectomy, while the Cancer and Leukemia Group B trial (CALGB140503) conducted by the Alliance for Clinical Trials in Oncology in North America, confirmed the non-inferiority of limited resection, including wedge resection for NSCLC measuring ≤2 cm. As both trials demonstrated non-inferiority of limited resection in NSCLC ≤2 cm, their results are often summarized together. However, patient background, radiological findings, prognosis, and extent of resection differ significantly between the two trials and should be interpreted with caution. Previous trials have demonstrated that preserving lung parenchyma helps maintain pulmonary function and improves patient prognosis by enabling appropriate management of subsequent malignancy or other diseases. Limited resection, including segmentectomy, is currently the standard of care for early-stage NSCLC. The JCOG and WJOG are conducting trials to determine whether the indications for limited resection can be expanded to include patients with NSCLC >2 cm or those with stage I NSCLC. This review article outlines the results of previous trials, provides an overview of ongoing trials, and discusses prospects for limited resection.

## Introduction

In 2021, a landmark study that significantly reshaped the surgical strategy for early-stage lung cancer was published [1]. The Lung Cancer Surgical Study Group (LCSSG) of the Japanese Clinical Oncology Group (JCOG) and West Japan Oncology Group (WJOG) (JCOG0802/WJOG4607L) trial demonstrated that, with appropriate patient selection, segmentectomy was superior to lobectomy for peripheral non-small cell lung cancer (NSCLC) ≤2 cm. One year after the publication of JCOG0802/WJOG4607L, the Cancer and Leukemia Group B trial (CALGB140503) also confirmed the efficacy of limited resection, including both segmentectomy and wedge resection, compared with lobectomy, although the patient population differed between the two studies [2]. Moreover, two confirmatory single-arm phase III prospective trials, the JCOG1211 trial and the JCOG0804/WJOG4507L trial, demonstrated the efficacy of limited resection in early-stage NSCLC with ground-glass opacity (GGO) predominance [3, 4]. This series of prospective trials has fundamentally changed the management and influenced the perspectives of thoracic surgeons.

Although limited resection is preferable to lobectomy for early-stage NSCLC, certain issues must still be addressed and resolved. This review discusses the history, clinical evidence, ongoing clinical trials, and prospects of limited resection of early-stage NSCLC.

### History and contemporary role of lobectomy and limited resection in lung cancer surgery

Lung resection has been reported since the 19^th^ century, with Graham describing the first successful case of one-stage pneumonectomy for carcinoma of the bronchus in 1933 [5]. Subsequently, Cahan et al. reported the definition of “radical pneumonectomy” in 1951, as the excision of the lung in continuity with its regional lymph nodes located in the hilar and mediastinal areas [6]. Cahan described lesser resection of one or two lobes of an entire lung with their specific regional hilar and mediastinal lymphatics as “radical lobectomy” in a study published in 1960 [7]. This procedure has remained the standard concept in lung cancer surgery for over half a century, despite the limited evidence regarding the optimal extent of resection [8].

Segmental resection or segmentectomy, with the excision of a smaller unit of the lobe, was developed for the management of local benign lung conditions [9, 10] and was initially introduced to “compromised” patients after the concept of radial lobectomy had been established. In 1972, LeRoux presented 17 cases of bronchial carcinoma that underwent segmental resection for various reasons [11]. In 1973, Jensik et al. reported detailed outcomes from a series of 123 patients who underwent segmentectomy for lung cancer [12]. Thus, limited resections such as segmentectomy initially emerged as a “compromised” procedure for patients unable to tolerate lobectomy for various reasons, including poor pulmonary function, age, and comorbidities. Wedge resection is a less invasive method compared with segmentectomy, as the procedure does not involve hilar structures and preserves the lung parenchyma. However, long-term oncologic control of wedge resection is considered inversely proportional to this lower surgical invasiveness.

### First randomized controlled trial (RCT) of lobectomy versus limited resection for stage IA NSCLC

Although limited resection was initially developed to avoid lobectomy in compromised patients, some investigators have argued that it may be appropriate for patients with T1N0 NSCLC who are otherwise healthy [13, 14]. This evidence, supported by retrospective studies, led the North America Lung Cancer Study Group (LCSG) to conduct a historic prospective multi-institutional randomized trial (LCSG821 trial) comparing the outcomes of limited resection and lobectomy for stage I lung cancer [15]. Between 1982 and 1988, a total of 276 patients with clinical stage IA NSCLC were enrolled, and 247 were randomized. As a primary endpoint, the limited resection group, including those undergoing wedge resection and segmentectomy, had inferior overall survival (OS) compared to those in the lobectomy group, with 5-year OS rates of 42% and 63%, respectively (one-sided P = 0.088). Furthermore, the local recurrence rate in the limited resection group was approximately three times that in the lobectomy group, which is reasonably high and does not prove the efficacy and validity of limited resection for clinical stage IA NSCLC. However, critical challenges, including radiological imprecision, inaccurate clinical staging, and small sample sizes, must be considered. In addition, some debate exists that non-anatomical wedge resection may result in worse survival rates than segmentectomy [16, 17], however, wedge resection has been performed in a substantial proportion of patients. Following this trial, no large-scale studies have compared lobectomy with limited resection for lung cancer. Thus, lobectomy has long been considered the standard procedure for NSCLC.

### JCOG clinical trials for peripheral early-stage NSCLC

Despite the inferior survival rate associated with limited resection in the aforementioned prospective RCT, many retrospective studies have indicated that segmentectomy may achieve oncological outcomes comparable to lobectomy [18–23]. After the LCSG821 trial, high-resolution computed tomography (CT) was introduced to detect small-sized lung cancers, and thin-section CT (TSCT) revealed features of less invasive GGO-dominant lung cancers. A prospective radiological study based on TSCT (JCOG0201) revealed that the consolidation-to-tumor ratio (CTR) and total tumor diameter are key predictors of less invasive lung cancer [24]. Based on the results of this trial, the JCOG and WJOG conducted three prospective studies stratified by tumor size and CTR [1, 4, 25].

JCOG0802/WJOG4607L multicenter, open-label, phase III non-inferiority RCT was conducted to evaluate whether segmentectomy was non-inferior to lobectomy in patients with clinical stage IA peripheral NSCLC characterized by tumor diameter ≤2 cm and CTR >0.5 [1]. A total of 1106 patients were enrolled to undergo lobectomy (n = 554) or segmentectomy (n = 552) between 2009 and 2014. The 5-year OS rates were 94.3% and 91.1% for segmentectomy and lobectomy, respectively, meeting the primary endpoint by confirming the non-inferiority of segmentectomy in OS (hazard ratio [HR]: 0.663; 95% confidence interval [CI]: 0.474–0.927; one-sided p<0.0001) and also demonstrating its superiority (p = 0.0082). The local recurrence rate for segmentectomy was nearly double that observed after lobectomy (10.5% vs. 5.4%); however, the 5-year recurrence-free survival (RFS) rate was similar in both groups (segmentectomy, 88.0%; lobectomy, 87.9%). Thus, this clinical study has provided new evidence by demonstrating the benefit of segmentectomy as a limited resection method for peripheral stage IA NSCLC, even when more than half of the tumor is a consolidated or invasive component.

JCOG0804/WJOG4507L single-arm confirmatory trial first demonstrated the efficacy of limited resection for radiologically less-invasive or non-invasive lung cancer with a maximum tumor diameter of 2.0 cm or less and a CTR of 0.25 or less, based on the TSCT defined by JCOG0802 [4]. Wedge resection served as the primary surgical approach, while segmentectomy was performed when adequate surgical margins could not be achieved with wedge resection. Of the 314 patients, 258 (82%) underwent wide-wedge resections and 56 (18%) underwent segmentectomies. The 5-year RFS rate was 99.7% (90% CI, 98.3–99.9), meeting the primary endpoint, without local recurrence.

Based on the favorable prognosis of GGO-dominant tumors, a multicenter, single-arm, confirmatory phase III trial (JCOG1211) was conducted to evaluate the efficacy of segmentectomy in patients with GGO-dominant NSCLC ≤3 cm [3]. Of the 357 patients who underwent segmentectomy, the primary endpoint of 5-year RFS was 98.0% (95% CI 95.9–99.1), exceeding the pre-specified 5-year RFS threshold of 87%. Here, segmentectomy as limited resection proved to be the standard treatment for patients with a GGO-dominant NSCLC ≤3 cm, with three prospective phase III trials in Japan providing evidence supporting limited resection for peripheral early-stage NSCLC based on TSCT findings.

### CALGB140503 trial for peripheral early-stage NSCLC

In North America, the Alliance for Clinical Trials in Oncology conducted the phase III RCT, CALGB140503, for peripheral NSCLC measuring ≤2 cm, during approximately the same period as JCOG0802/WJOG4607L [2]. Patients were enrolled if the tumor was ≤2 cm with a solid component, and pathologically confirmed pN0 NSCLC either preoperatively or intraoperatively. The primary endpoints of this study were disease-free survival (DFS) and the non-inferiority of sublobar resection (wedge resection or segmentectomy) over lobectomy. A total of 697 patients were enrolled and randomized to undergo sublobar resection (n = 340) or lobectomy (n = 357) between 2007 and 2017. The results revealed that sublobar resection was non-inferior to lobectomy in terms of DFS (HR, 1.01; 90% CI, 0.83–1.24). The 5-year DFS rates were 63.6% and 64.1% in the sublobar resection and lobectomy groups, respectively. The OS in both groups was similar, and no significant difference was observed in the incidence of locoregional or distant recurrence between the two groups. The CALGB140503 trial further validated the efficacy of limited resection outside Japan, and these RCTs have redefined the standard of care for peripheral small NSCLC.

### Difference between JCOG0802/WJOG4607L and CALGB140503

Although both JCOG0802/WJOG4607L and CALGB140503 trials [2] demonstrated non-inferiority for NSCLC ≤2 cm, their results are often summarized together; however, patient background, radiologic findings, prognosis, and extent of resection differ significantly between the two trials. The most significant differences were in surgical extent in the limited group, intraoperative confirmation of negative pathological lymph node metastasis, and patient selection based on radiological findings (Table 1).

**Table 1:** Differences in study design between JCOG0802/WJOG4607L and CALGB140503 trials.

	JCOG0802/WJOG4607L	CALGB140503
Organization	JCOG/WJOG	Alliance for Clinical Trials in Oncology/NCI
Study design	RCT, non-inferiority	RCT, non-inferiority
Primary endpoint	Overall survival	Disease-free survival
Experimental arm	Segmentectomy only	Sublobar resection (Segmentectomy:37.9%/wedge resection:59.1%)
Lymph node status	cN0	pN0 confirmed
Lymph node dissection	Systematic or selective lymph node dissection was mandatory	Intraoperative or preoperative examination of both superior and subcarinal nodes
Target tumor size	≤2cm	≤2cm
CTR	0.5<CTR≤1	Not specified
Accrual	Completed	Sample size down due to slow accrual
Number of patients	1106 (lob arm = 554, seg arm = 552)	697 (lob arm = 357, seg arm = 340)
Study start date	Aug. 2009	Jun. 2007
Final registration	Oct. 2014	Mar. 2017

RCT, randomized controlled trial; JCOG, Japanese Clinical Oncology Group; WJOG, West Japan Oncology Group; NCI, National Cancer Institute; CALBG, Cancer and Leukemia Group B; CTR, consolidation to tumor ratio

First, only segmentectomy was performed in the JCOG0802/WJ-OG4607L trial, whereas more than half of the patients in the sublobar resection group underwent wedge resection in the CALGB140503 trial. In the latter trial, the pathological N0 status was confirmed intraoperatively in the hilar and mediastinal nodes, when preoperative nodal evaluation had not been performed. In contrast, the JCOG0802/WJOG4607L trial required systematic or selective lymph node dissection; however, intraoperative frozen section diagnosis was not always performed. Fundamentally, the terms “limited resection” and “sublobar resection” are used interchangeably to refer to resections smaller in scale than lobectomy, encompassing wedge resection and segmentectomy. However, the concepts of “limited resection” or “sublobar resection” differ between Japan and North America. Second, JCOG0802/WJOG4607L included part-solid ground-glass nodules (GGN) with a predominantly solid appearance, whereas CALGB140503 did not specify the presence of GGO. In relation to this radiological finding, over 90% of patients in the JCOG0802/WJOG4607L trial had adenocarcinoma, compared with fewer than two-thirds of patients in the CALGB140503 trial (Table 2). Regarding patient outcomes, although the non-inferiority of limited resection was confirmed in both trials, the 5-year OS rate for CALGB140503 was >10% lower in both the lobectomy and limited resection groups than in the JCOG0802/WJOG4607L group, while the 5-year RFS rate was >20% lower in both procedures than in the JCOG0802/WJOG4607L group. Similarly, local recurrence rates were approximately 10% higher for each procedure, and the overall recurrence rates were approximately 15–20% higher in the CALGB trial.

**Table 2:** Differences in outcome between JCOG0802/WJOG4607L and CALGB140503 trials.

	JCOG0802/WJOG4607L				CALGB140503	
	All		Only solid			
	Lobectomy	Segmentectomy	Lobectomy	Segmentectomy	Lobectomy	Sublobar resection
Median age (years)	67	67	67	66	68	68
Male (%)	52.9	52.5	62.0	63.4	41.2	44.1
PS0/PS1/PS2 (%)	97.7/2.3/0	98.2/1.8/0	97.8/2.2/0	97.8/2.2/0	70.0/28.6/1.4	77.4/21.2/1.5
Never smoked (%)	44.4	44.2	33.6	33.7	9.8	8.2
Ad/ Sq/ Others (%)	90.4/6.9/2.7	90.9/6.7/2.4	79.9/9.5/10.6	77.8/12.5/9.7	63.3/14.8/21.8	64.1/13.2/22.6
pN0/pN1/pN2	94.2/2.9/2.7	93.5/3.1/3.1	90.5/5.1/4.4	88.8/5.0/6.1	100/0/0	100/0/0
Change of FEV1.0 at 6month post-op (%)	-13.1	-10.4	-	-	-6.0	-4.0
5-y OS rate (%)	91.1	94.3	86.1	92.4	78.9	80.3
HR of OS (95% CI)	1	0.663 (0.474–0.927)	1	0.64 (0.42–0.97)	1	0.95 (0.83–1.24)
5-y RFS/DFS rate	87.9	88.0	81.7	82.0	64.1	63.6
HR of RFS/DFS	1	0.998 (0.753–1.323)	1	1.01 (0.72–1.42)	1	1.01 (0.83–1.24)
Any recurrence (%)	7.9	12.1	12.4	18.6	29.8	30.6
Local recurrence (%)	3.1	6.9	7.7	16.1	12.7	15.3

JCOG, Japanese Clinical Oncology Group; WJOG, West Japan Oncology Group; CALBG, Cancer and Leukemia Group B; OS, overall survival; HR, hazard ratio; CI, confidence interval; RFS, recurrence-free survival; DFS, disease-free survival; FEV1.0, forced expiratory volume in 1 s; post-op, postoperative

One possible explanation for the difference in survival between the two trials may be the patient selection based on CT findings and the proportion of part-solid GGN. However, differences in OS and RFS persisted even after selecting patients with NSCLC exhibiting a radiologically pure solid appearance (Table 2) [2, 26]. Although direct comparisons between different patient populations from separate trials are not possible, the poorer prognosis observed in the CALGB 140503 trial may be attributed to a higher proportion of patients with poor performance status, non-adenocarcinoma histology, and a history of smoking. We believe that wedge resection has a worse impact on survival than segmentectomy due to poorer local control. Wedge resection may fail to provide adequate surgical margins particularly in the central side compared with segmentectomy. Furthermore, even with extensive pathological examination of mediastinal lymph nodes in the CALGB140503 trial, hilar lymph node dissection may be insufficient. However, unexpectedly, post-hoc analysis of CALGB140503 demonstrated no statistical differences between wedge resection and segmentectomy in terms of OS, DFS, or incidence of locoregional recurrence [27]. Caution is required when interpreting the difference between wedge resection and segmentectomy. Regarding the cause of death, both trials reported nearly equivalent lung cancer-specific mortality rates for lobectomy, limited resection, and segmentectomy. Notably, in the JCOG0802/WJOG4607L trial, the non-lung cancer mortality rate in the lobectomy group (9.4%) was twice that in the segmentectomy group (4.9%), leading to a significant difference in OS.

One of the advantages of limited resection is the preservation of postoperative lung function [28, 29]. Interestingly, both the JCOG0802/WJOG4607L and CALGB140503 trials favored limited resection in terms of the reduction in predicted forced expiratory volume in 1 s (FEV1.0) at 6 months postoperatively; however, the difference between lobectomy and limited resection was only 2–3%. In the JCOG0802/WJOG4607L trial, the median decrease in FEV1.0 at 1 year postoperatively for segmentectomy was 3.5% less than that for lobectomy and did not reach the predefined threshold for clinical significance of 10%. However, even when accounting for the results of previous retrospective studies, establishing a 10% threshold may have been challenging. Given the benefit of the OS of limited resection, parenchymal preservation may contribute not only to modest improvements in pulmonary function but also to less obvious advantages, such as enhanced oxygen uptake, improved exercise tolerance, and reduced cardiac burden. Future studies are needed to elucidate the prognostic impact of the number of resected segments and the extent of parenchymal preservation on their subtle physiological effects, as well as to evaluate the benefits of additional treatment for recurrent or secondary malignancies.

### Technical considerations of segmentectomy

“Limited resection” included segmentectomy and wedge resection. First, whether these techniques have the same surgical difficulty and prognostic impact is debatable. Wedge resection is a straightforward procedure; however, segmentectomy is technically more challenging. Furthermore, segmentectomy is a relatively simple procedure involving the left upper, lingual, and superior (S6) or basilar segments of the lower lobes [30, 31]. Conversely, complex segmentectomy requires transecting the lung parenchyma from multiple directions and dissecting surrounding structures deep within the lung [32]. Surgical quality is also a critical factor for segmentectomy. Approximately 20% of segmentectomies were reported to be performed as “non-anatomical” procedures, in which at least one major vascular structure or segmental bronchus is not divided or dissected [33]. Non-anatomic segmentectomies may lead to a poor patient prognosis due to smaller margins, fewer lymph nodes, and limited sampling of mediastinal lymph node stations.

The surgical approach is technically important. Currently, 80% of the segmentectomies in Japan are performed using video-assisted thoracoscopic surgery (VATS) [34]. Although few large-scale studies have examined the long-term postoperative survival rates of VATS, robot-assisted thoracoscopic surgery (RATS), or open thoracotomy—and prospective comparative studies remain challenging—VATS and RATS offer potential advantages. These minimally invasive approaches allow detailed visualization of anatomical structures from multiple angles and may be associated with reduced acute pain and a lower incidence of chronic numbness [35, 36].

### Ongoing clinical trials of limited resection in NSCLC

**Figure 1:**
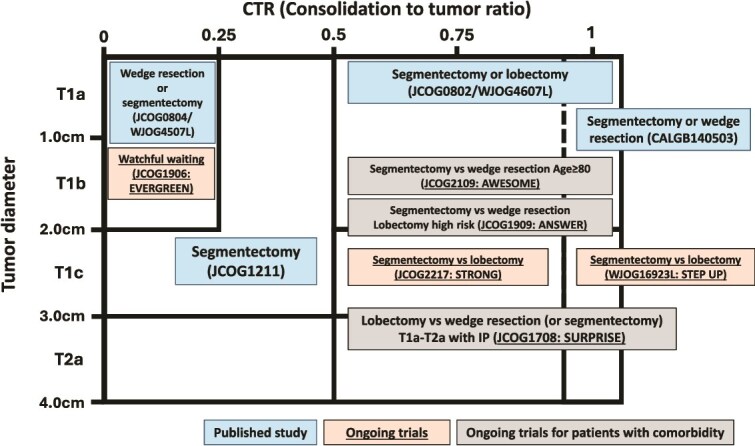
Ongoing prospective clinical trials in Japan conducted by JCOG and WJOG according to tumor diameter and CTR. JCOG, Japan Clinical Oncology Group; WJOG, West Japan Oncology Group; CTR, consolidation-to-tumor ratio.

In Japan, the JCOG and the WJOG are conducting various prospective clinical trials for NSCLC based on the results of previous clinical trials (Figure 1). Two RCTs are being conducted to expand the indications for segmentectomy for NSCLC measuring 2–3 cm. One was WJOG16923L (STEP UP trial) for pure-solid NSCLC with clinical stage IA3 (2–3 cm) [37], and the other was JCOG2217 (STRONG trial) for patients with radiological solid-predominant GGN with clinical stage IA2-IA3 (2–3 cm).

Three RCTs comparing limited resection are ongoing to examine the efficacy of limited resection in patients with various comorbidities, including interstitial pneumonia and advanced age. The JCOG2109 (AWESOME trial) is a phase III RCT comparing segmentectomy with wedge resection in older patients (≥80 years) with peripheral solid or solid-dominant NSCLC ≤2 cm who are considered suitable candidates for lobectomy. The JCOG1708 (SURPRISE trial) evaluated a phase III RCT comparing lobectomy with sublobar resection (wedge resection or segmentectomy if wedge resection was impossible) in patients with idiopathic pulmonary fibrosis and clinical T1a-2aN0M0 peripheral solid- or solid-dominant NSCLC [38]. JCOG1909 (ANSWER trial) is a phase III RCT comparing segmentectomy and wedge resection in patients with peripheral solid or solid-dominant NSCLC ≤3 cm who are deemed high-risk candidates for lobectomy [39].

The JCOG1906 (EVERGREEN trial) [40] is a distinctive prospective trial evaluating the efficacy and safety of watchful waiting to determine the optimal timing of surgical intervention in patients with radiologically GGO-dominant non-invasive lung cancers with a low risk of metastasis—the same population studied in JCOG0804/WJOG4507L. Outside Japan, two active surveillance studies have been conducted (TSOG102 [41] and ECTOP-1021). These studies will demonstrate the actual trends and need for resection in early-stage, non-invasive GGO-dominant lung cancer.

Table 3 lists ongoing national and international clinical trials on limited resection and active surveillance of early-stage NSCLC. In China, the indications for limited resection according to the presence or absence of micropapillary and solid subtypes based on frozen section diagnosis have also been investigated (STAR 001 and STAR 002). The results of these prospective studies warrant expanding the indications of limited resection.

**Table 3:** Ongoing clinical trials involving limited resection and active surveillance worldwide.

Trial	Clinical trial Number	Region	Objectives	Procedure	Progress	Planned accrual	Accrual initiation	Primary endpoint
**GGO-predominant tumor**								
TSCI 002	NCT02718365	China	0.5cm<GGO≤2.0cm and CTR≤ 0.25	Segmentectomy vs wedge resection	Completed	1382	12/2017	5-year DFS
TSOG102	NCT03802981	North America	0.6≤GGO≤3.0cm and CTR<0.5	Active surveillance	Completed	330	1/2019	5-year LCSS
JCOG1906 (EVERGREEN)	UMIN000040818	Japan	GGO≤2cm and CTR≤0.25	Active surveillance	Recruiting	500 †	6/2020	10-year OS
ECTOP-1019	NCT06031181	China	AIS/MIA diagnosed by Intraoperative Frozen Section	Sublobar Resection	Recruiting	390	3/2023	5-year RFS
ECTOP-1020	NCT06102161	China	GGO≤2cm and a CTR:0.25-0.5	Wedge resection (single arm)	Not yet recruiting	286	11/2023	5-year OS
ECTOP-1021	NCT06097910	China	0.6cm≤GGO≤2cm and CTR<0.25	Active surveillance	Recruiting	290	11/2023	5-year OS
**Tumor with GGO ≤3cm**								
GREAT	ChiCTR20000-37065	China	0.5cm<GGO≤3cm, with 0.5cm< consolidation ≤2cm (cT1a-b)	Segmentectomy vs Lobectomy	Recruiting	1024	1/2021	5-year RFS
ECTOP-1012	NCT05717803	China	2cm≤GGO≤3cm and CTR<0.5	Segmentectomy	Recruiting	277	2/2023	5-year DFS
JCOG2217 (STRONG)	jRCT1030240027	Japan	2.0<GGO≤3.0cm and CTR≥0.5	Segmentectomy vs Lobectomy	Recruiting	490^†^	4/2024	5-year OS
SELTIC	NCT06646770	Turkey	2.0<GGO≤3.0cm and CTR≥0.5	Segmentectomy vs Lobectomy	Not yet recruiting	400	1/2025	5-year OS and RFS
**Tumor ≤2cm**								
201212107RIND	NCT03185754	Taiwan	NSCLC ≤ 2cm	Sublobar resection vs Lobectomy	Recruiting?	600	6/2013	5-year RFS
STAR 001	NCT04937283	China	LUAD ≤ 2cm without MPP and solid subtype	Segmentectomy vs Lobectomy	Recruiting	690	10/2019	5-year RFS
STAR 002	NCT05838053	China	LUAD ≤ 2cm with MPP and solid subtype (>5%)	Segmentectomy vs Lobectomy	Recruiting	446	8/2019	5-year RFS
2021-SR-164	NCT04944563	China	NSCLC ≤ 2 cm in the Middle Third of the Lung Field	Segmentectomy vs Lobectomy	Recruiting	1120	7/2021	5-year DFS
**Tumor ≤3cm**								
WJOG16923L (STEP UP)	UMIN000052064	Japan	Pure-solid NSCLC with clinical T1cN0	Segmentectomy vs Lobectomy	Recruiting	520†	1/2024	5-year OS
**Patient with comorbidity**								
CTONG1504	NCT02360761	China	Older patients (≥70) with cT1N0M0 (≤3cm)	Sublobar resection vs Lobectomy	Recruiting?	339	1/2016	3-year RFS
JCOG1708 (SURPRISE)	UMIN000032696	Japan	Stage I NSCLC patients with IPF	Sublobar resection vs Lobectomy	Recruiting	430†	5/2018	5-year OS
JCOG1909 (ANSWER)	UMIN000040089	Japan	High-risk patients for lobectomy with stage IA	Segmentectomy vs Wedge resection	Recruiting	330†	4/2020	5-year OS
JCOG2109 (AWESOME)	jRCT1030220482	Japan	Older patients (≥80) with NSCLC ≤2cm, CTR>0.5	Segmentectomy vs wedge resection	Recruiting	320†	12/2022	5-year OS

GGO, ground-glass opacity; TSOG, Thoracic Surgical Oncology Group; NCT, National Clinical Trial; CTR, consolidation to tumor ratio; LCSS, lung cancer–specific survival; JCOG, Japan Clinical Oncology Group; UMIN, University Hospital Medical Information Network; OS, overall survival; ECTOP, Eastern Cooperative Thoracic Oncology Project; ChiCTR, Chinese Clinical Trial Registry; RFS, Recurrence-free survival; jRCT, Japan Registry of Clinical Trials; LUAD, lung adenocarcinoma; MPP, micropapillary; WJOG, West Japan Oncology Group; NSCLC, Non-small cell lung cancer; CTONG, Chinese Thoracic Oncology Group; IPF, Idiopathic pulmonary fibrosis; † for second registration

### Current concerns and future directions

As described above, evidence for limited resection in early-stage lung cancer has been established, and clinical trials are currently underway to further expand this evidence according to patient conditions. However, the concept of “limited resection” differs between Japan and North America, and whether these differences can be reconciled remains unclear. In the JCOG trial, CTR was employed to select patients; however, whether this concept can be applied globally as an indication for limited resection remains unclear.

JCOG0802/WJOG4607L excluded NSCLC with pathologically positive lymph nodes from limited resection, and CALGB140503 confirmed pN0 for inclusion; however, patients with occult lymph node metastases after second registration were included in JCOG0802/WJOG4607L, and the limitations of intraoperative frozen diagnosis should also be considered. The optimal extent of lymph node dissection during segmentectomy remains controversial. When a patient with clinical N0 disease undergoes segmentectomy and has occult lymph node metastases postoperatively, the necessity for additional lobectomy to ensure optimal resection remains debatable.

All tumors included in the clinical trials were peripheral NSCLC located in the outer one-third of the lung. Although lobectomy is standard for centrally located NSCLC, segmentectomy may be considered for non-peripheral tumors amenable to complete resection. The JCOG plans to initiate a new prospective trial, “JCOG2501; UNLIMITED Trial”, comparing lobectomy and segmentectomy for centrally located NSCLC.

Nonsurgical treatments, such as radiotherapy, are also evolving. Future efforts should focus on identifying optimal treatment for individual patients with early-stage lung cancer, including strategies for follow-up and observation. As technology evolves, we must consider the next steps in using next-generation tools, such as artificial intelligence, to select appropriate personalized treatments. In the management of early-stage cancer, not only curative treatment, but also true oncological outcomes, including quality of life, must be considered.
